# An overview of artificial intelligence in oncology

**DOI:** 10.2144/fsoa-2021-0074

**Published:** 2022-02-10

**Authors:** Eduardo Farina, Jacqueline J Nabhen, Maria Inez Dacoregio, Felipe Batalini, Fabio Y Moraes

**Affiliations:** 1 Department of Radiology, Federal University of São Paulo, SP, 04021-001, Brazil; Diagnósticos da America SA (Dasa), 05425-020, Brazil; 2 School of Medicine, Federal University of Paraná, Curitiba, PR, 80060-000, Brazil; 3 School of Medicine, State University of Centro-Oeste, Guarapuava, PR, 85040-167, Brazil; 4 Department of Medicine, Division of Medical Oncology, Beth Israel Deaconess Medical Center, Harvard Medical School, Boston, MA 02215, USA; 5 Department of Oncology, Division of Radiation Oncology, Queen's University, Kingston, ON, K7L 3N6, Canada

**Keywords:** artificial intelligence, cancer diagnosis, data integration, medical oncology, patient stratification, precision oncology

## Abstract

Cancer is associated with significant morbimortality globally. Advances in screening, diagnosis, management and survivorship were substantial in the last decades, however, challenges in providing personalized and data-oriented care remain. Artificial intelligence (AI), a branch of computer science used for predictions and automation, has emerged as potential solution to improve the healthcare journey and to promote precision in healthcare. AI applications in oncology include, but are not limited to, optimization of cancer research, improvement of clinical practice (eg., prediction of the association of multiple parameters and outcomes – prognosis and response) and better understanding of tumor molecular biology. In this review, we examine the current state of AI in oncology, including fundamentals, current applications, limitations and future perspectives.

## Plain language summary

Cancer is associated with significant morbimortality globally. Although significant advances occurred in the last decades, challenges in providing personalized care remain. Artificial intelligence (AI) has emerged as a mean of improving cancer care using compure science. AI applications in oncology include, but are not limited to, optimization of cancer research, improvement of clinical practice (including prediction of cancer patients outcomes and response to treatment) and better understanding of tumor characteristics. In this review, we explored the current state of AI in oncology, including fundamentals, current applications, limitations and future perspectives.

Cancer accounts for significant morbidity and mortality worldwide. An estimated 19.3 million new cancer cases occurred in 2020 [1], and this figure is expected to increase over the next few decades. Projections show that 30.2 million new cancer cases will be diagnosed in 2040 [1].

Despite substantial improvements in cancer diagnosis and management [2] that have resulted in a reduction of cancer mortality over the last two decades, a staggering 10 million cancer-related deaths occurred in 2020 [1]. It is imperative to promote innovation in healthcare and especially in cancer care.

Early diagnosis of cancers remains a major global challenge. Effective screening initiatives are limited by public buy-in, financial support, etc. and do not cover all at-risk populations [3]. However, expanding screening initiatives without evidence-based indication can lead to a significant financial burden and waste valuable resources in resource-constrained health systems [4].

Although cancer treatment options have expanded in the last decades, only a subset of privledged patients benefit from novel cancer drugs and the cost-benefit ratio of current treatments is suboptimal [4]. Thus, there is an urgent need to make cancer treatment more affordable and personalized.

The development of new anticancer treatments is a time and resource-intensive process. Even after a drug passes preclinical testing and undergoes clinical trials, the success rate is low, and patient enrollment becomes challenging [5]. Despite these challenges, 64 interventions focused on cancer diagnostic or treatment were approved or had their indications expanded by the US FDA in 2020 [6]. The fast-paced environment of cancer research leads to a surplus of relevant literature posing a challenge to physicians trying to apply the latest recommendations to their practice.

Data captured from oncology providers and healthcare systems are complex and diverse. Doctors' typed or dictated notes, laboratory findings, histopathological and imaging data and patient-generated health data are examples of the unpredictability of the information captured. Crude medical data are of often of limited relevance, thus obtaining meaningful clinical insights and analytics relies on adequate data extraction, processing, analysis, interpretation and integration.

Acknowledging that the capacity of the human brain to process information is limited, there is an urgent need for the implemantation of alternative strategies to process modern big data (describes the large volume of data – both structured and unstructured – that inundates a healthcare on a day-to-day basis). In addition to the increased availability of data, the augmentation of storage and computing power has boosted the development of data-processing techniques, such as machine learning (ML) and artificial intelligence (AI), which are becoming increasingly important tools to tackle complex issues in cancer care. A growing body of studies highlight AI as an emerging tool to help personalize cancer-care strategies by analyzing available data. A recent study identified 97 registered clinical trials for AI in cancer diagnosis, most of them started after 2017 [7].

In this narrative review, we provide an overview of the role of artificial intelligence in oncology, including current applications, future perspectives and limitations.

## Artificial intelligence

Aritficial intelligence can be described as a branch of computer science dealing with the simulation of intelligent behavior in computers. It relies on computers following algorithms established by humans or learned by computer method to support decisions or execute certain tasks [8]. Machine learning is a subfield of AI and represents the process by which a computer is able to improve its own performance by continuously incorporating newly-generated data into an existing iterative model [9]. Deep learning (DL) is a subfield of ML where mathematical algorithms are deployed using multi-layered computational units resembling human cognition. These include neural networks with differente architetures types (e.g., recurrent neural networks, convolutional neural network and long term short memory).

Artificial neural networks may have different architecture on how they apply mathematical rules to data and can be useful to analyze unstructured data [10]. Unstructured data are a very common type of medical data used to record qualitative and subjective information typically acquired through patient–provider interactions or imaging acquisition. Applying AI to unstructured text data can be achieved by natural language processing (NLP) techniques and recurrent neural networks are DL algorithms commonly useful for this task. In contrast, convolutional neural networks are the most used and promising AI architectures in the exploration of imaging files.

**Figure 1.**
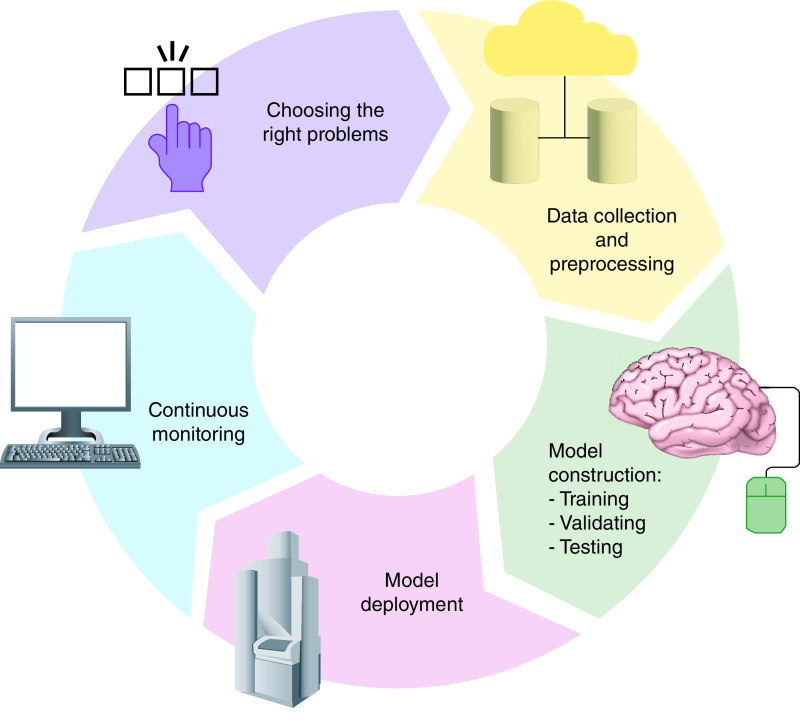
Artificial intelligence flywheel. Graphic representation of the artificial Intelligence and data cycle for building effective and responsible machine learning models for healthcare.

The development and validation of ML models include the the correct problem, data collection, pre-processing (e.g., anonymization), training, internal validation, testing, optimization, evaluation and finally, external validation [11]. Every step is important to create a reliable machine learning model that can be applied into clinical practice. After the deployment of any model, results and application should be constantly monitored for drift checking – loss of performance – to ensure model consistency (Figure 1). Moreover, the clinical utility of ML models must be assessed in prospective clinical trials using specific metrics defined for each problem. The most commonly used metric used for classifications tasks in medicine is the receiver operating characteristic curve (ROC curve). ROC curve plots the true positive rate and false positive rate and the area under the ROC curve (AUROC) expresses the level of accuracy. In addition, the confusion matrix is used to assess sensitivity, specificity and precision (Table 1) [12,13].

**Table 1.** Artificial intelligence and precision oncology glossary.

Terms	Definitions
Algorithm	A set of rules for solving a problem or for performing a task
Area under curve	A measure of a classifier's accuracy for a binary classification
Artificial intelligence	Systems that display intelligent behavior by analyzing their environment and taking actions – with some degree of autonomy – to achieve specific goals
Artificial neural network	A computional model in machine learning, which is inspired by the biological structures and functions of the human brain
Computer-aided detection/diagnosis	Systems that use computer science to assist doctors in the interpretation of medical images
Deep learning	A subfield of machine learning that mimics the capacity of the human brain to perform unsupervised learning tasks using multiple layers of neural networks
Machine learning	A field in computer science that builds computational models that have the ability of ‘learning’ from data and providing predictions
Radiomics	A method that extracts and analyses large amounts of advanced quantitative image features with the intent of creating mineable databases from radiological images
Radiogenomics	A field that studies the correlation between cancer imaging features and gene expression

This table represents a summary of terms used in the areas of artificial intelligence combined with precision oncology [13].

## Artificial intelligence for cancer imaging

Artificial intelligence is particularly applicable in medical fields that deal with images, notably radiology and pathology [14]. In radiology, there are many applications of AI, especially DL algorithms to analyze imaging data acquired during routine cancer care including disease classification, detection, segmentation, characterization and monitoring [15,16].

- Classification: image classification is necessary in cancer screening studies. AI can help radiologists achieve better outcomes, save time and support the classification of small lesions. It can also help on the creation of a better organizational workflow (e.g., determining a high priority group of reports to be reviewed and reported). There are examples of studies showing that combining AI and human power improved mammography screening for breast cancer [17,18]
- Detection: AI can aid in the identification of cancerous lesions that could otherwise be missed by humans. For instance, it can be used to find lung nodules [19] or brain metastases on MRI readouts [20]. Detection relies on the the use of bounding boxes to detect a lesion or object of interest. Detection using AI supports physician on their process of reading medical images (i.e., lung nodules) [21].
- Segmentation: helps to classify individual pixels according to organs or lesions by precisely recognizing lesions and accessing its volume and size. For example, brain gliomas require quantitative metrics for their management, risk stratification and prognostication [22]
- Characterization: deep learning methods can be applied to medical images to extract a large number of features undetectable by humans, and potentially uncover disease characteristics and patterns. Radiomics is the field that studies these features and there is growing interest in combining these features with clinicogenomic information. Radiomics methods can inform models that successfully predict treatment response and/or side effects from cancer treatments [23]. There is a variety of cancer types where radiomics can be applied such as liver, brain, and lung tumors [24,25]. Deep learning using radiomic features from brain MRI has the ability to differentiate brain gliomas from brain metastasis with similar performance to trained neuroradiologists [26]
- Monitoring: the aforementioned techniques can be used to monitor a particular lesion (e.g. stability vs progression). Using AI can change dramatically the way cancer is monitored because it has the ability to detect a multitude of discriminative features in imaging unreadable by humans [15]

Generative adversarial networks (GANs) are AI models that can generate new images based on any type of data. A possible application is the generation of synthetic computed tomography (CT) imaging of from MRI imaging. This technology has the potential to support radiotherapy planning [27]. Additionally, it has proven useful in automating dose distribution for intensity modulated radiation therapy (IMRT) for prostate cancers [28].

Also, generative networks, including additional types of architectures (e.g., autoencoders [AEs] and variational autoencoders [VAEs]), have the capability of improving the acquisition of multimodality imaging, such as MRI and CT scans, reducting radiation dose and use of intravenous contrast [29–31]. Since oncology patients must do routine scans for tumor staging, AE and VAE have the potential to reduce healthcare costs while improving patient safety.

Additionally, deep learning models can be used to predict future development cancer. The concept of care gap is that eventually patients do routine scans or MRI for other conditions and some AI models already have been developed to predict disease, for instance cardiovascular scores from CT scans [32,33]. A study reported on the ability to predict a 5-year future breast cancer risk from normal mammograms using deep-learning CNNs [34]. Predicting future cancer from a normal scan is promising and is armed to have a great populational impact.

AI models can also be applied to pathology and photographs. Golatkar *et al.*, reported that a deep learning model based on convolutional neural networks exhibited over 90% accuracy of in classifying benign versus malignant histology from hematoxylin and eosin (H&E) stained breast biopsy samples [35]. Dermoscopic images have been used to classify lesions as benign or malignant and were able to reach the same accuracy level as trained dermatologists [36].

Currently, some of the AI applications are already being implemented in clinical practice [37–39]. Further development, refinement and application of AI to real-world data is warranted. Such goal can only be achieved with a trained workforce which underscores the urgency of the education of the next generation of physician-scientists in AI and oncology [40].

## Artificial intelligence for predicting clinically relevant parameters

Exploring of the vast data captured by electronic health records (EHRs) has allowed investigators to identify patterns of clinically relevant parameters using individual and historical data as aggregated data [41]. EHRs organized data in a standard structure, which can be processed using AI-based natural language processing algorithms. These can be a cost-effective and straightforward tool to support medical decision making. The deep patient representation is an example of the automated use of patient data from large-scale EHR databases to predict desired outcomes [41]. In this model, raw EHR information was processed through multiple layers of neural networks to allow clinically-relevant analyses such as disease development risk [41]. The applicability of such models in real life settings requires overcoming obstacles such as data standardization, technological infra structure and organizational data culture.

**Figure 2.**
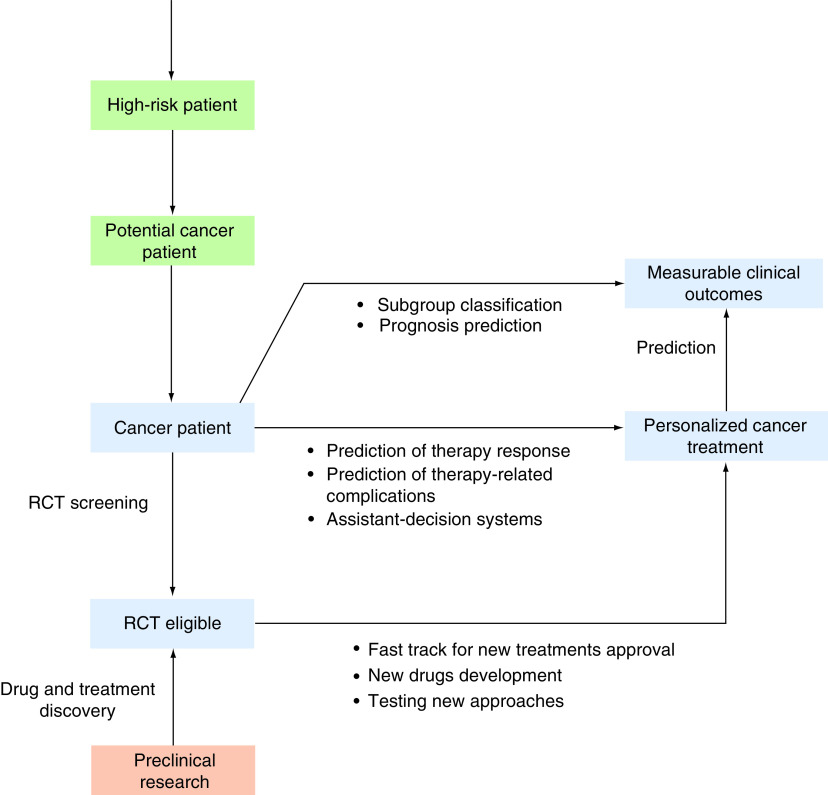
Potential applications of artificial intelligence in a cancer patient's journey. AI-based models can be used in preclinical (orange box) and in clinical scenarios, both before and after cancer diagnosis (green and blue boxes, respectively). In real-life oncology care, AI has the potential to optimize risk stratification, screening recommendations, diagnosis, prognosis, decision-making and treatment-related outcome prediction. Connecting clinical research to routine oncology practice by efficient drug repurposing, accelerated new treatment discovery and efficient patient matching to RCTs is another potential contribution of AI. AI: Artificial intelligence; RCT: Randomized controlled trial.

Medical imaging can also be a source of prognostic information. Radiomics can be applied to assess and predict clinically relevant parameters in oncology [42]. Due to imaging being routinely performed for cancer diagnosis and patient follow up, radiomics could theoretically be easily integrated in cancer care. Other types of information, such as genomic data, can also be used for prognostic purposes [43]. Risk-stratification, treatment complications, survival, and therapy response are some of the prognostic parameters that can be accessed using AI algorithms (Figure 2). But there is still a long road ahead and education of stakeholders is also a key factor for success.

### Risk stratification

A well-known application of EHR data is disease risk stratification. Calculating risk stratification was limited by the quantity of data that could be retrospectively reviewed, and analyzed using traditional statistical methods. Artificial intelligence-based algorithms have proven to be able to assess unstructured data and accurately estimate the probability of patients developing various diseases including cancer [41]. Agnostic AI models can refine risk-stratification definitions and impact decisions on cancer screening recommendations [44–49] with satisfactory accuracy. For example, an artificial neural network model for colorectal cancer risk stratification showed improved accuracy when compared with current screening guidelines, by reducing false positives (i.e., individuals misclassified as high risk) from 53 to 6% and false negatives (i.e., individuals misclassified as low risk) from 35 to 5% [45].

These AI models could be used on a populational scale. High-risk individuals not included in the current screening guidelines but who are still at high risk for cancer development would likely be identified and benefit from early assessment. For example, screening for patients with early-onset sporadic colorectal cancer is limited by traditional methods, but may potentially benefit from intensive risk-based screening recommendations [45].

Individuals who are at low risk for cancer development despite being included in current screening recommendations would be able to choose not to be screened which would impact the system opportunity cost (opportunity cost is an economics term that refers to the loss of potential benefits from other options when one option is chosen) [50]. This would not only allow a shared doctor–patient decision-making process but also could relieve the system from inefficient and harmful interventions.

For tumors with no established screening approach which are mainly asymptomatic at initial stages, personalized risk-prediction could facilitate early diagnosis and potentially lead to higher cure rates. For example, an artificial neural network model for pancreatic cancer risk prediction has achieved an area under the ROC curve of 85% [47]. Algorithms for personalized risk-calculation can help prioritize screening for high-risk individuals in low-resource settings.

### Treatment complications

AI has the potential to predict treatment-related toxicity related to radiation [51] and chemotherapy [52,53]. This has the potential to guide the discussion of risks and benefits associated with different treatment modalities and support personalized RT dose-delivery.

ML models have been able to predict visit to emergency rooms and hospital admissions due to cancer therapy-related symptoms [54]. Using those predictions in clinical practice can help with the provision of a preventive supportive approach to high-risk patients. This would not only improve patient care but also relieve healthcare systems with the burden of preventable hospital encounters.

### Survival & disease recurrence

Algorithms for survival prediction have been developed for many cancer types, including breast, prostate and lung cancers [55–58]. AI-based algorithms have shown better accuracy for predicting survival than conventional analytic approaches [58]. This may be because they have improved fit for variables with nonlinear relationships, and thus are more applicable to real-life settings. Predicting cancer survival can help tailor treatment strategies. Treatment planning can be reinforced for patients at high risk while interventions with marginal benefit for low-risk patients could be avoided [55]. In addition, the risk of disease reccurence after curative treatment can been predicted using AI models. The use of AI for recurrence prediction has showed increased accuracy compared with conventional statistical models [59], which will further support clinical follow-up plan optimization.

### Therapy response

AI can help predicting treatment response [60–62] using tumor characteristics obtained from radiologic images. Individual patient responses to high-cost treatments such as immunotherapy can be predicted [61] and may help in-patient care decision-making, and facilitate efficient use of healthcare resources. Prediction of complete pathological response after neoadjuvant treatments [62] could reduce treatment intensity since it allows identification of patients who would be candidates for a conservative approach rather than radical interventions. Algorithms using pharmacogenomics to predict individual treatment response have also been developed [43].

## Artificial intelligence for cancer diagnosis

Cancer diagnoses can also be optimized using AI. AI-powered colonoscopy has shown to be a cost-effective intervention by efficiently identifing benign polyps thus not requiring resection [63]. This would not only save healthcare resources but would also prevent adverse events from a more invasive treatment approach. Accurate diagnosis of cancerous and precancerous lesions can allow for minimization of overtreatment. On that note, AI algorithms supporting colposcopic images evaluation have shown high accurary in predicting precancerous lesions in cervical cancer screening [64]. AI-based precise cancer stratification at diagnosis can help in minimazing invasive interventions and unnecessary surgical procedures [65].

Identifying molecular features without the need for high-cost genetic testing is another application of AI. AI-based algorithms have shown efficacy in predicting microsatellite instability by analysis of common hematoxylin and eosin (H&E) stained tissue slides [66,67]. Low-cost and integrated analysis of this biomarker could be used to support use of immunotherapy in select cases and identify at-risk families.

## Artificial intelligence for cancer research

Recent studies have pointed out that the benefits of AI in cancer care go beyond optimization of current established treatment strategies. AI is also applicable in preclinical settings such as basic / translational research and cancer drugs development [68]. Artificial intelligence can help integrate and process information from multiple databases and enable drug repurposing [69]. AI identifies potential new drugs within a short time period at an affordable cost [69]. Drug testing can simulate and predict the effectiveness of cancer therapies leading to better results in *in vivo* experiments [70], which in turn would accelerate clinical research.

Clinical trials can also become more efficient with the use of AI. Study outcomes can be predicted using AI models [71] which could significantly lower costs of drug development. AI has been used to identify patients for clinical trials [72] by incorporating inclusion and exclusion criteria to search EHR and identify eligible patients, hence facilitating participant accrual. These systems have shown high accuracy while only requiring a fifth of the time used by manual review [73]. Previously published data suggested that a higher rate of clinical trial enrollment not only leads to faster advances in cancer treatment but is also related to better cancer population survival outcomes [74].

## Artificial intelligence & personalized medicine

Many innovations in oncology patient care have been due to the large amount of information derived from patients' individual biological and clinical characteristics (i.e., genomics, radiomics, metabolomics and other ‘-omics’) and the development of biomarkers, targeted therapies, imaging technologies and wireless monitoring devices. AI has emerged as an instrument to help physicians to deliver more precise and accurate care [75]. Recommendations generated by its immense data analysis capabilities can be useful in delivering personalized medicine. There are a number of processes that AI can have a substantial impact including cancer prevention, drug discovery and genomic-based interventions [76].

In molecular biology, AI is promoting unique insights and improvements on tumor biology understanding through the collaboration of biological and computer scientists [77].

Cancer is a disease of the genome, so it's no wonder that oncology has particularly benefited from AI innovations. For instance, DNA methylation assessment in cancers has been proven to be useful for classification and prognostication [78]. The machine-determined DNA methylation approach can lead to the recategorization of more than 70% of human-labeled tumors, which could lead to significantly different prognostication and treatment decisions [79].

In a seminal study from Capper D *et al.* [80], whole-genome methylation analysis of tumor specimens using the Illumina HumanMethylation450 (450 k) or MethylationEPIC (850 k) array platforms was shown to have 93% accuracy in classifying 82 classes of brain tumors. The accuracy reported by the authors far exceeded the accuracy of pathologists.

Assistant-decision systems, such as Watson for Oncology, have shown acceptable concordance with the decisions made by multidisciplinary teams. This can can aid in patient-level decision making in a fast and less resource-intensive manner [81]. Furthermore, new algorithms that predict waiting time to cancer surgery are allowing a personalized pre-rehabilitation approach [82] that could potentially result in better surgical outcomes.

AI systems offer accurate data and image analyses, but results are only useful if validated, interpretable and clinically relevant. A successful incorporation of AI-based systems into clinical practice requires training of the intended users and basic education on the methods to all stakeholders, including its limitations and ethical dilemmas [83,84]. AI models also promise to be valuable in complex cases such as in those patients who present as cancer of unknown primary, which still represents 1–2% of newly diagnosed cancers [85]. A deep learning model based on H&E-stained whole-slide imaging was able to classify the site of origin of metastatic tumor with 83% accuracy [86]. Technologies like this are particularly valuable since most patients do not have access to extensive characterization of their tumors.

AI's role in precision oncology is evident; it can enhance human capabilities by enable the incorporation of increasingly complex knowledge into clinical decision making. It facilitates the interpretation of the increasingly of diverse and complex data and its application for personalized management.

## AI from lab to clinics: challenges & scopes

Despite AI-based algorithms having been implemented by many corporations for data evaluation, their translation into clinical practice remains a challenge [87]. Barriers include limitations in data collection and training, scarcity of prospective clinical validation, difficulties in user education and ethical/regulatory guidelines [88,89]. Challenges related to data range accuracy to relevancy of the information assembled. Meaningful data needs to be relevant, with high quality and processable [90].

The first step for data analysis is the pre-processing of a defined set(s) of data(s). This requires normalization, noise filtering and feature selection when more than one dataset is combined. Normalization becomes an essential step to eliminate bias when analyzing different sets of data that are merged. The selection of defined features is a critical phase in the success of a classification, regression and pattern recognition algorithm. Another major challenge in precision oncology is to integrate data generated from various types of omics and multiple sources of information to predict biomarkers or clinical outcomes [90].

In addition, there is a relative ignorance of the medical community related to AI and its methods and applications. Education of all stakeholders including patients, providers and business administrators is necessary so that advances can be translated into a higher quality care [40,83,91]. A seamless integration of any new tool into clinical workflow is critical to its long-term success. Rigby et al. highlighted the ethical challenge with AI in healthcare. It is imperative to address the ethical issues related to use of patient data in unwarranted and unconsented circumstances while respecting ethical policies and guidelines designed to protect patient safety and privacy [84].

Although AI can be employed to lower costs in the several scenarios presented in this review, significant infrastructure investments are required to enable its application. Data storage and compute power are not free of cost, and human resources (including information technology and bioinformatics personnel) are important for the timely and consistent application of these tools [92]. Cloud services are becoming more widespread and could potentially decrease the need for initial investments on single-institution high-performance computing clusters and dedicated professionals. Nonetheless, storage costs and compute time still incur significant expenses, and reimbursement for AI-based clinical services will have to be defined. Quality control processes will need to be in place to ensure safe application of technology [93]. It is necessary to point out, however, that although AI development and implementation costs may pose a challenge, initial investment translates into significant process enhancement at minimal additional future costs [87].

## Conclusion

AI has already had a significant impact in healthcare and will continue to revolutionize medicine. The potential is tremendous and has applications in cancer research, screening, diagnosis, treatment and monitoring. AI also has the potential to decrease healthcare costs and disparities. Several tools have been developed harnessing the diverse set of medical data (including free-text, laboratory and imaging results, radiological images and omics data). With these goals in mind, further research is necessary to continue and ensure analytical and clinical validity and clinical utility.

## Future perspective

Once challenges are addressed and AI algorithms are validated by prospective studies, the future direction of AI-based models is to be a part of healthcare in every single scenario. In the near future, oncology AI applications will happen through data intelligence, better tumor understanding, more precise treatment options and improved decision-making processes [94]. Oncology will become a more precise speciality and patients will be move than ever at the center of care [94].

In addition, risk assessment tools incorporated to smarthphone applications will provide an immediate cancer risk estimation for the general public. Patients who receive high risk estimatives can be motivated to seek for medical care and to adhere to medical recommendations. Also, estimatives of risk reduction can motivate individuals toward the improvement of personal habits such as quitting smoking or engaging into physical activity. In primary care settings, algorithms will help physicians to decide when to refer patients to high-complexity health centers. Healthcare centers can be benefited from algorithm incorporation into EHR systems as an alternative for better allocation of resources (based on the knowledge of the subgroup of patients that has higher risk of cancer development, or cancer-related complications).

**Executive summary**

- Artificial intelligence (AI) essentials: main concepts about AI are discussed in this part to enable a better comprehension of the article for healthcare workers.
- Artificial intelligence for cancer imaging: current applications of AI in oncology imaging and future perspectives on how it can impact even more healthcare.
- Artificial intelligence for predicting clinically relevant parameters: how AI is enabling better understanding of individual patients, such as risk factors, treatment complications, therapy response and survival.
- Artificial Intelligence for cancer diagnosis: examples of AI-powered tools that are improving cancer diagnosis accuracy.
- Artificial intelligence for cancer research: how AI can reduce costs and time in cancer research such as drug discovery and patient selection for clinical trials.
- Artificial intelligence and personalized medicine: cases whereas AI can improve personalized medicine from molecular and genomics to a more broad perspective.
- Limitations and future perspectives: a summary from the limitations and future impacts of the previously discussed applications.
